# The fate of methylmercury through the formation of bismethylmercury sulfide as an intermediate in mice

**DOI:** 10.1038/s41598-021-96579-y

**Published:** 2021-09-02

**Authors:** Yumi Abiko, Yusuke Katayama, Wenyang Zhao, Sawako Horai, Kenji Sakurai, Yoshito Kumagai

**Affiliations:** 1 Faculty of Medicine, University of Tsukuba, 1-1-1 Tennodai, Tsukuba, Ibaraki 305-8575 Japan; 2 Master’s Program in Medical Sciences, Graduate School of Comprehensive Human Sciences, University of Tsukuba, Tsukuba, Ibaraki 305-8575 Japan; 3 Doctoral Program in Materials Science and Engineering, Graduate School of Pure and Applied Sciences, University of Tsukuba, Tsukuba, Ibaraki 305-0006 Japan; 4 National Institute for Materials Science, Tsukuba, Ibaraki 305-0047 Japan; 5 Environmental Health Section, Department Environment and Public Health, National Institute for Minamata Disease, Minamata, Kumamoto 867-0008 Japan

**Keywords:** Metals, Environmental sciences, Metabolic pathways

## Abstract

A previous study by our group indicated that methylmercury (MeHg) is biotransformed to bismethylmercury sulfide [(MeHg)_2_S)] by interaction with reactive sulfur species (RSS) produced in the body. In the present study, we explored the transformation of MeHg to (MeHg)_2_S in the gut and the subsequent fate of (MeHg)_2_S *in vitro* and *in vivo*. An *ex vivo* experiment suggested the possibility of the extracellular transformation of MeHg to (MeHg)_2_S in the distal colon, and accordingly, the MeHg sulfur adduct was detected in the intestinal contents and feces of mice administered MeHg, suggesting that (MeHg)_2_S is formed through reactions between MeHg and RSS in the gut. In a cell-free system, we found that (MeHg)_2_S undergoes degradation in a time-dependent manner, resulting in the formation of mercury sulfide and dimethylmercury (DMeHg), as determined by X-ray diffraction and gas chromatography/mass spectrometry, respectively. We also identified DMeHg in the expiration after the intraperitoneal administration of (MeHg)_2_S to mice. Thus, our present study identified a new fate of MeHg through (MeHg)_2_S as an intermediate, which leads to conversion of volatile DMeHg in the body.

## Introduction

Mercury species are naturally occurring substances and industrially produced environmental contaminants, and their biogeochemical cycle is well known. For example, volatile mercury species, such as Hg^0^ or inorganic Hg^2+^, are deposited on soil and in water bodies by rainfall during volcanic activity. Inorganic mercury is methylated to yield methylmercury (MeHg) and then undergoes further methylation to form dimethylmercury (DMeHg) through nonenzymatic reactions and/or biotransformations in microbes^1^. MeHg in the environment can accumulate in fishes, such as tuna (*Thunnus*), and other marine creatures^1,2^. Humans are mainly exposed to MeHg through the intake of contaminated foods that are health risks. Due to the electrophilic nature of MeHg, this organomercury compound covalently modifies protein thiols, thus leading to the formation of MeHg-protein adducts, which change protein functions^3,4^. We previously found that a lower MeHg dose modifies Kelch-like ECH-associated protein 1 (Keap1) and phosphatase and tensin homolog deleted on chromosome ten (PTEN), thus leading to the activation of the Keap1/nuclear factor E2 related factor 2 (Nrf2) pathway and PTEN/Akt signaling, which are related to detoxication of xenobiotics and cell survival, respectively, whereas MeHg at a high dose disrupts cellular homeostasis, resulting in cell death^5,6^. Unlike MeHg, DMeHg has little electrophilicity for modifying protein thiols but shows high and delayed toxicity^7,8^.

**Figure 1:**
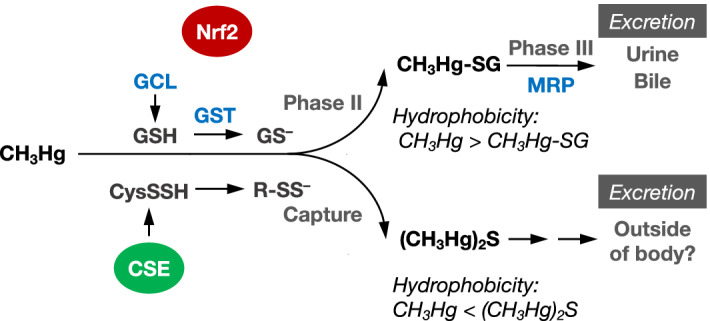
Biotransformation of methylmercury in the body. *CSE* cystathionine γ-lyase, *CySSH* cysteine persulfide, *GCL* glutamate-cysteine ligase, *GSH* glutathione, *GS*^*–*^ deprotonated form of GSH, *GST* GSH *S*-transferase, *CH*_*3*_*Hg-SG* methylmercury GSH adduct, *MRP* multidrug resistance associated protein.

After being incorporated into the body, MeHg is effectively absorbed from the gastrointestinal tract and distributed through blood to various organs. Some MeHg in tissues interacts with small molecular nucleophiles, such as glutathione (GSH) produced by glutamate-cysteine ligase (GCL), in the absence and presence of GSH *S*-transferase (GST), and these interactions are referred to as phase II reactions and result in the formation of GSH adducts, which are excreted into the extracellular space through multidrug resistance-associated protein (MRP) in phase III reactions^9^. The series of reactions regulated by Nrf2 are considered a strategy to remove hydrophobic MeHg from cells (tissues) by conversion to a more hydrophilic GSH adduct (Fig. 1) because this transcription factor cooperatively regulates the gene expression of GCL, GST and MRP^10,11^. Although this polar GSH conjugates a detoxified metabolite^9,12^, the Hg–S bond on the MeHg–SG adduct is unstable^13,14^. Supporting this, our study suggested that MeHg–SG adduct undergoes *S*-transmercuration with reactive CysSH residues on proteins, resulting in formation of protein-MeHg adducts^15^, which are associated with the activation of redox signaling pathways and toxicity^3,4^, as mentioned above. Moreover, we previously identified bismethylmercury sulfide [methyl(methylmercuriosulfanyl)mercury; (MeHg)_2_S] as a metabolite of MeHg from the liver of rats given MeHg and neuroblastoma SH-SY5Y cells exposed to MeHg^16^. Subsequent examination indicated that (MeHg)_2_S is formed by the interaction of MeHg with reactive sulfur species (RSS), which exhibit high nucleophilicity^17,18^ and include compounds such as GSH persulfide (GSSH) and protein-bound persulfide derived from CysSH persulfide (CysSSH) and hydrogen sulfide (H_2_S) produced by transsulfuration or enzymatic activities, such as cystathionine γ-lyase (CSE) activity^19^ (see Fig. 1). Although gut microbiota can produce H_2_S under anaerobic and aerobic conditions^20–22^, the transformation of MeHg to (MeHg)_2_S in gut is unknown. Unlike the MeHg–SG adduct, (MeHg)_2_S cannot interact with protein thiols^16^, suggesting that this sulfur adduct no longer has the covalent binding capability involved in MeHg toxicity. Nevertheless, (MeHg)_2_S is more hydrophobic than the parent compound MeHg. Therefore, we hypothesized that a system is responsible for excreting the sulfur adduct of MeHg outside the body. In the present study, we explored (1) the biotransformation of MeHg to (MeHg)_2_S in the gut and (2) the fate of (MeHg)_2_S *in vitro* and *in vivo*.

## Results

### Ex vivo and in vivo studies on the biotransformation of MeHg

**Figure 2:**
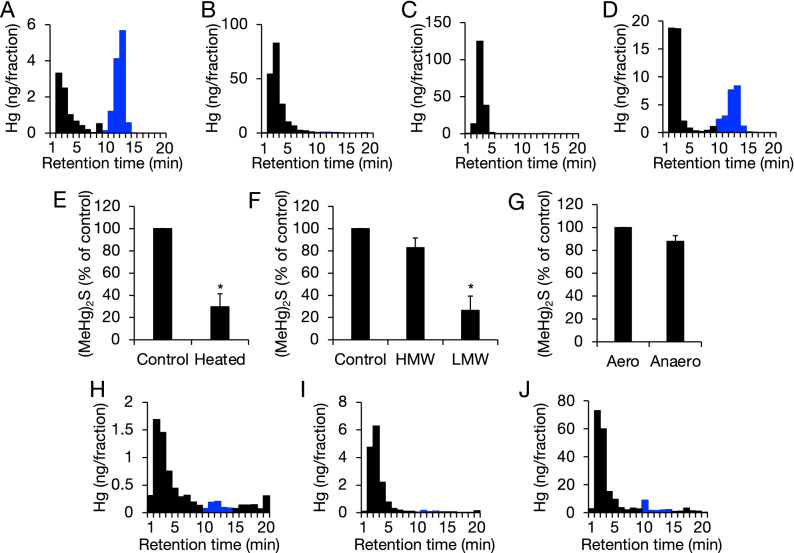
Detection of bismethylmercury sulfide as a reaction product of methylmercury with intestinal content *in vitro* and *in vivo*. Blue bars indicate (MeHg)_2_S. MeHg (200 µM) was incubated in mouse (**A**) distal colon or (**B**) duodenum with content, (**C**) distal colon without content, or (**D**) 10 mg of the content for 30 min. (**E**) MeHg (200 µM) was incubated with 10 mg of the content, which was preincubated at 25 °C (control) or 95 °C (heated) for 15 min, for 30 min. Each value of (MeHg)_2_S is the mean ± SE of three determinations. *P < 0.05 vs. control. (**F**) MeHg (200 µM) was incubated with 50 mg/mL homogenized content (control), its high-molecular-weight fraction (HMW), or its low-molecular-weight fraction (LMW) for 30 min. The control sample was incubated in a tube under atmospheric air. Each value of (MeHg)_2_S is the mean ± SE of three determinations. *P < 0.05 vs. control. (**G**) MeHg (200 µM) was incubated with homogenized intestine contents (50 mg/mL) for 30 min under anaerobic conditions (anaero). The control sample was incubated under atmospheric air (aero). Each value of (MeHg)_2_S is the mean ± SE of three determinations. After 18 h of a single treatment with MeHg (10 mg/kg, p.o.), (**H**) the distal colon and (**I**) duodenum contents were collected. (**J**) The feces from mice given MeHg (10 mg/kg, p.o.) was collected after 18 h–42 h of exposure. The samples were analyzed by HPLC/AAS. Representative data are shown.

First, we determined whether MeHg reacted with RSS in intestinal lumen to form (MeHg)_2_S. (MeHg)_2_S was produced by the incubation of MeHg (200 µM) in the mouse distal colon (but not in the duodenum with the colon’s natural content) for 30 min under aerobic conditions (Fig. 2A,B), and a peak containing Hg with a retention time of 10–14 min was observed by high-performance liquid chromatography (HPLC)/atomic absorption spectrophotometry (AAS)^19^. Although (MeHg)_2_S was not detected during incubation of MeHg when the distal colon was washed with saline (Fig. 2C), incubation of MeHg with even the content alone produced (MeHg)_2_S (Fig. 2D). We boiled the intestinal contents to determine the contribution of proteins in microbes to (MeHg)_2_S formation and then reacted them with MeHg. As a result, (MeHg)_2_S formation in the reaction mixture with boiled contents was decreased to 30% of the control (Fig. 2E). Correspondingly, using the supernatant of intestinal contents after removing the high-molecular-weight fraction significantly decreased the formation of (MeHg)_2_S from MeHg compared with using the whole supernatant (Fig. 2F). Moreover, significant differences in (MeHg)_2_S production were not observed when the intestinal content and MeHg were incubated under aerobic or anaerobic conditions (Fig. 2G).

MeHg (10 mg/kg) was orally injected into mice, and (MeHg)_2_S formation in the gut was analyzed. As shown in Fig. 2H–J, (MeHg)_2_S was detected in the distal colon content and fecal sample but not in the duodenum content. These results suggest that fecal excretion of (MeHg)_2_S is, at least partially, attributable to the formation of (MeHg)_2_S from MeHg by RSS produced by the distal colon microbiota. Such a formation of (MeHg)_2_S from MeHg was also seen in the rectal content of another mammal, the small Indian mongoose (Supplemental Fig. S1).

### Product analysis during degradation of (MeHg)_2_S

**Figure 3:**
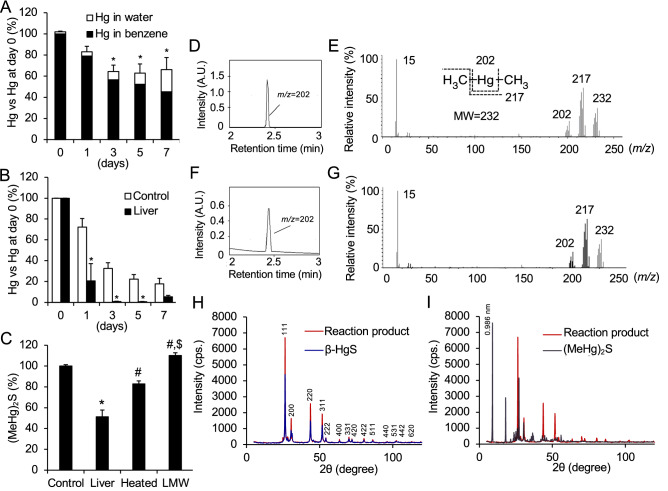
Detection of organic and inorganic mercury compounds during incubation with bismethylmercury sulfide. (**A**) (MeHg)_2_S was incubated for 0–7 days at 37 °C. After liquid–liquid extraction, the benzene layer and the water layer were separately analyzed by AAS. Each value is the mean ± SE of three determinations. *P < 0.01 vs. 0 day. (**B**) (MeHg)_2_S was incubated in mouse liver supernatant (liver) isolated by centrifugation at 9,000*g* or in 50 mM KPi (pH 7.5) (control) for 0–7 days at 37 °C and detected by HPLC/AAS. Each value is the mean ± SE of three determinations. *P < 0.01 vs. 0 day. (**C**) Mouse liver 9000*g* supernatant (2 mg/mL) was preincubated at 25 °C or 95 °C. (MeHg)_2_S (100 µM) was incubated in 50 mM KPi (pH 7.5) (control), supernatant (liver), heated supernatant (heated), or low-molecular-weight fraction of mouse liver 9000*g* supernatant in 50 mM KPi (pH 7.5) (LMW) for 1 day at 37 °C. (MeHg)_2_S was analyzed by HPLC-AAS. Each value is the mean ± SE of three determinations. *P < 0.001 vs. control, ^#^*P* < 0.01 vs. liver, ^$^*P* < 0.01 vs. heated. The head space of a sample tube containing (MeHg)_2_S was collected and analyzed by GC/MS. (**D**) Chromatogram of authentic DMeHg and (**E**) spectrum of the peak with a retention time of 2.5 min. (**F**) Chromatogram of the collected compounds and (**G**) spectrum of the peak with a retention time of 2.5 min. The residue after 7 days of incubation with (MeHg)_2_S was purified. (**H**, **I**) Decomposition product (reaction product), (**H**) authentic β-HgS, and (**I**) (MeHg)_2_S were analyzed by X-ray diffraction, and the patterns are shown.

We tested the stability of (MeHg)_2_S at 25 °C in 50 mM potassium phosphate buffer (KPi, pH 7.5) for 7 days. (MeHg)_2_S in the sample, as detected by HPLC/AAS, was gradually degraded to 17.8% of the starting material, while MeHg was fairly stable under these conditions (Supplemental Fig. S2). We separated organic Hg species from the solution by liquid–liquid extraction with benzene and water to confirm the decomposition of (MeHg)_2_S and found that the Hg level in the benzene layer after 7 days was decreased to approximately 45.2% of the value at day 0, while the Hg level in the water layer increased in a time-dependent manner (Fig. 3A). In a separate experiment, (MeHg)_2_S was also incubated with mouse liver supernatant centrifuged at 9000*g*. Although 72.2% of (MeHg)_2_S in 50 mM KPi buffer remained after one day, (MeHg)_2_S in the 9000*g* supernatant markedly declined to 20.8% (Fig. 3B). Under this condition, heat treatment at 95 °C and the removal of the high-molecular-weight fraction of the 9000*g* supernatant significantly suppressed the degradation of (MeHg)_2_S compared to that with the nonheated supernatant (Fig. 3C), suggesting that heat-labile proteins participate in (MeHg)_2_S decomposition. Notably, the material balance of Hg levels was incomplete under these conditions (Fig. 3A), suggesting that unknown decomposition products seem to be volatile substances. To confirm this possibility, we collected unknown compounds from the head space of a sample tube containing (MeHg)_2_S by using XAD-4 resin for 3 h. Gas chromatography/mass spectrometry (GC/MS) revealed that a product derived from (MeHg)_2_S with a retention time of 2.5 min had *m/z* = 202 (^202^Hg) (Fig. 3F), which is identical to that of authentic dimethylmercury (DMeHg) analyzed by GC/MS (Fig. 3D). In addition, the spectrum of the unknown product showing the parent ion (*m/z* = 232) and the fragment ions (*m/z* = 217, 202, and 15) (Fig. 3G) were almost the same as that of authentic DMeHg (Fig. 3F).

Moreover, we also collected black particles in the aqueous phase of the sample after incubation for 7 days, and the crystal structures were analyzed by X-ray diffraction (XRD). As shown in Fig. 3H, the XRD pattern of the present black particles was nearly identical to that of authentic black β-HgS, which has a cubic structure (space group *F_43m*, a = 0.58537 nm, ICDD PDF card 01-089-0432^23^). The four strongest peaks were observed at 26.34°, 43.72°, 51.76°, and 30.52° for 111, 220, 311, and 200 reflections, respectively. A series of small peaks at 54.24°, 63.60°, 70.04°, 72.10°, 80.30°, 86.32°, 96.32°, 102.26°, 104.38°, and 112.80° were observed for 222, 400, 331, 420, 422, 511, 440, 531, 442, and 620 reflections, respectively^23^. Moreover, three or four small additional peaks were observed between 20° and 30° in the XRD patterns of the obtained black particles and authentic β-HgS (Fig. 3H,I). As discussed in Supplemental Fig. S3, all such XRD peaks were well explained by considering the second phase of α-HgS, which has a hexagonal structure (space group *P3*_*2*_*21*, a = 0.41495 nm, c = 0.9497 nm, ICDD PDF card 00-042-1408^24^). In contrast, in Fig. 3I, the XRD pattern of the present black particles was clearly different from that of (MeHg)_2_S itself because the strongest peak from (MeHg)_2_S at 9.00° (corresponding to 0.983 nm interplanar spacing) vanished completely. This finding clearly demonstrates that the original (MeHg)_2_S had decomposed and that an inorganic crystal of β-HgS was formed.

### Detection of CH_4_ during decomposition of DMeHg in vitro

**Figure 4:**
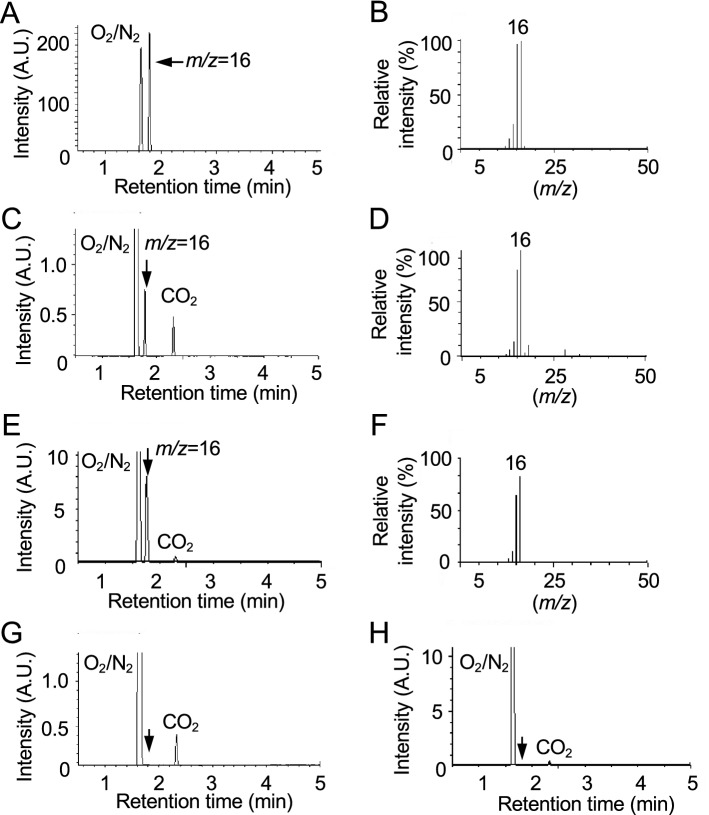
Detection of CH_4_ during dimethylmercury or methylmercury incubation by GC/MS analysis*.* The arrows indicate CH_4_. (**A**) Chromatogram of authentic CH_4_ and (**B**) spectrum of the peak with a retention time of 1.79 min. Compounds in the head space of DMeHg solution after (**C**, **D**) 3 h or (**E**, **F**) 4 days of incubation were analyzed by GC/MS. (**C**, **E**) Chromatogram of the collected compounds and (**D**, **F**) spectrum of the peak with retention time of 1.79 min. Chromatogram of the collected compounds in the head space of MeHg solution after (**G**) 3 h or (**H**) 4 days of incubation.

**Figure 5:**
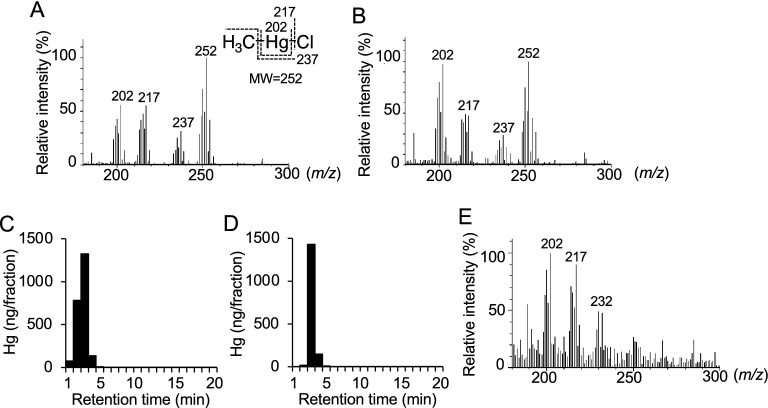
Detection of MeHg during incubation of dimethylmercury*.* MeHg or DMeHg were incubated in 0.5 N HCl-50% methanol for 4 days. EI-MS spectra of (**A**) authentic MeHgCl and (**B**) DMeHg. Hg levels of (**C**) MeHg and (**D**) DMeHg solution were detected by HPLC/AAS analysis. (**E**) EI-MS spectrum of DMeHg in methanol.

Because it is suggested that DMeHg is unstable under acidic conditions^25,26^, we incubated DMeHg or MeHg in 0.5 N HCl solution and analyzed the decomposition products. A peak with a retention time of 1.79 min corresponding to methane (CH_4_) (Fig. 4A,B) on GC/MS was detected in the head space of DMeHg at 3 h and 4 days of incubation (Fig. 4C–F), but not that of MeHg (Fig. 4G,H). This observation was consistent with previous findings^26^. Under the same conditions, a product with a similar electron ionization (EI)-MS fragmentation pattern as MeHgCl (Fig. 5A) was also observed in the DMeHg solution (Fig. 5B), and the peak containing Hg with the same retention time as authentic MeHg was detected by HPLC/AAS (Fig. 5C,D). We also analyzed DMeHg dissolved in methanol by EI-MS and observed DMeHg; however, the MeHg level was negligible (Fig. 5E). These results suggest that DMeHg decomposed into CH_4_ and MeHg in aqueous solution.

### Identification of DMeHg from the exhaled breath of mice

**Figure 6:**
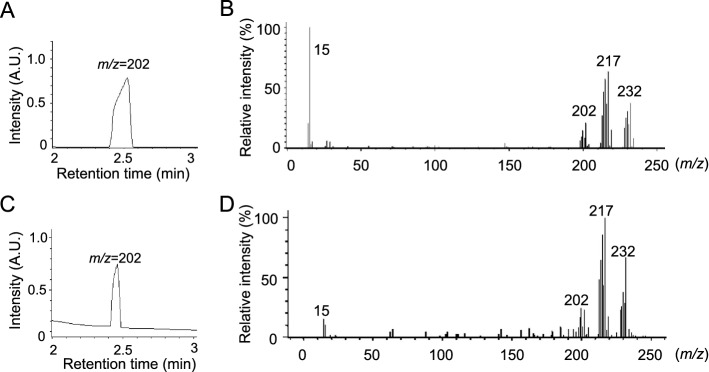
GC/MS analysis of mouse expirate after treatment with dimethylmercury or bismethylmercury sulfide*.* Chemicals in the exhaled air of mice given a single injection of (**A**, **B**) 0.1 mmol/kg DMeHg and (**C**, **D**) (MeHg)_2_S were analyzed by GC/MS. (**A**, **C**) Chromatogram of the collected compounds and (**B**, **D**) spectra of the peak with a retention time of 2.5 min. Representative data are shown.

Since DMeHg is a volatile material with a high vapor pressure (8.8 kPa at 20 °C, reference from ICSC), we confirmed whether DMeHg derived from (MeHg)_2_S was distributed in the lung and then exhaled outside of the body. To address this issue, DMeHg (0.1 mmol/kg) was intraperitoneally injected into mice and then the exhaled air was collected for 3 h. As shown in Fig. 6A,B, the collected sample showed a peak with a retention time of 2.5 min and an ion trace at *m/z* = 202 (^202^Hg), and the spectrum pattern corresponded to authentic DMeHg (Figs. 3D,E, 6A,B). The samples collected 3 h after the intraperitoneal administration of 0.01 mmol/kg (MeHg)_2_S or MeHg exhibited almost the same patterns as authentic DMeHg according to GC/MS (Fig. 6C,D, data not shown).

## Discussion

**Figure 7:**
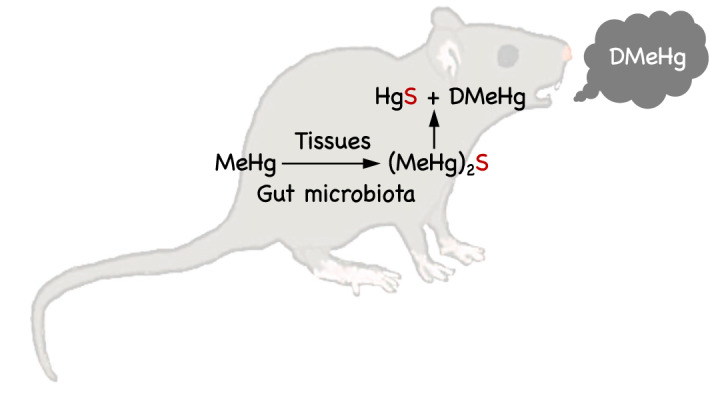
Excretion of dimethylmercury through the formation of bismethylmercury sulfide in mice. *DMeHg* dimethylmercury, *MeHg* methylmercury, *(MeHg)*_*2*_*S* bismethylmercury sulfide. MeHg was transformed into (MeHg)_2_S *via* reaction with reactive sulfur species in tissue and intestine. A portion of intestinal (MeHg)_2_S might be absorbed into the body. (MeHg)_2_S slowly decomposed to HgS and DMeHg, which could be excreted from the body.

In the present study, we identified DMeHg as a decomposition product of (MeHg)_2_S derived from MeHg *in vitro* and *in vivo*. Moreover, HgS was also formed during the decomposition of (MeHg)_2_S. In nonbiological samples, Craig and Bartlett previously observed biphasic decay of MeHg in the presence of H_2_S in an aqueous reaction mixture, with an initial rapid decay and then a slow decay that were probably due to the formation of (MeHg)_2_S, which is a somewhat water-insoluble material, and evolution of DMeHg from the reaction mixture, respectively^27^, thus supporting the results of our cell-free study. We performed a series of experiments and suggested that H_2_S generated in sediments may accelerate the transformation of MeHg to volatile DMeHg, which moves to the atmosphere^27–29^. To our knowledge, previous reports have not focused on the biotransformation of (MeHg)_2_S to DMeHg in mammals. The present study indicates that (MeHg)_2_S biologically produced from MeHg is a key intermediate in the production of DMeHg, which is released from the lungs in mice (Figs. 6 and 7). Such (MeHg)_2_S degradation was facilitated by the addition of the mouse liver supernatant centrifuged at 9000*g*, suggesting that unidentified high-molecular-weight components contribute, at least in part, to the decomposition of (MeHg)_2_S.

We initially reported that (MeHg)_2_S was formed during interactions with RSS, such as H_2_S produced by cystathionine β-synthase (CBS) in SH-SY5Y cells and in rats^16^. However, a subsequent study indicated that CBS and CSE catalyze the transformation of cystine to CysSSH, which interacts with GSH, thus forming GSSH and H_2_S based on an equilibrium reaction among them^17^. In addition, we found that (MeHg)_2_S is formed with not only H_2_S but also GSSH, GSSSG, protein-bound RSS and the synthetic polysulfide Na_2_S_4_ during reaction with MeHg^19^. We have recently shown that phytochemical components in garlic contain RSS that form (MeHg)_2_S from MeHg^30^. Therefore, simultaneous intake of fish that accumulate MeHg, such as tuna, with garlic would potentially lead to the increased formation of (MeHg)_2_S and concomitant production of DMeHg; as a result, the accumulation of MeHg in the body may be repressed by the intake of garlic. Further study is required to elucidate this possibility *in vivo*.

In addition to various enzymes (e.g., CBS and CSE) in organs, H_2_S is produced by numerous gut microbes, such as *Clostridium*, *Desulfovibrio*, *Escherichia*, *Klebsiella*, *Salmonella*, *Streptococcus*, and *Enterobacter*^20–22^. H_2_S can react with oxidized thiol (sulfenic acid) compounds, including protein thiols, to yield persulfides, which have higher nucleophilicity than the corresponding monosulfides^31^. Although the gastrointestinal tract absorbs approximately 90% of MeHg^32^, we assumed that MeHg reacts with RSS to form (MeHg)_2_S derived from the bacterial system in the mouse intestine. Herein, we show for the first time that (MeHg)_2_S is produced in the mouse distal colon with its contents (Fig. 2). We also detected (MeHg)_2_S in the distal colon content and in mouse feces following MeHg administration (Fig. 2H–J). Interestingly, the low-molecular-weight fraction of supernatant from the intestinal content produced less (MeHg)_2_S than the high-molecular-weight fraction or the whole supernatant. While the LC/MS analysis indicated that H_2_S as well as H_2_S_2_ were observed in the mouse feces and that a small amount of H_2_S and H_2_S_2_ were in the germ-free mice^33^, our present study suggested that not only low-molecular-weight RSS but also protein-bound RSS in the intestinal content facilitate (MeHg)_2_S production from MeHg. Hence, it seems likely that the formation of (MeHg)_2_S in the distal colon content and substantial excretion of this sulfur adduct in mice are attributable to gut microbe-dependent RSS. In the present study, we also detected (MeHg)_2_S from the rectal content of the wild small Indian mongoose (*Herpestes auropunctatus*)(Supplemental Fig. S1), which has a relatively high level of total Hg in its tissues^34^ that did not undergo artificial exposure to MeHg, suggesting that (MeHg)_2_S formation, at least in part, is due to a biotransformation mediated by gut microbe-dependent RSS in the mongoose.

We incubated DMeHg with H_2_O and found that CH_4_ and MeHg were formed from DMeHg under acidic conditions (Figs. 4 and 5), suggesting that sulfur adducts of MeHg are critical precursors for (1) excretion from the body through the formation of volatile DMeHg and (2) substantial preservation of these parent substances in the body by recycling MeHg. In the present study, HgS was also identified by XRD during spontaneous degradation of (MeHg)_2_S, suggesting that HgS is the counterpart for DMeHg, although we did not examine the exact stoichiometry of (MeHg)_2_S and that these products formed in the study. Since HgS is an insoluble nanoparticle, the simple question of how this particle is stored or excreted from the body should be addressed in the near future.

## Methods

### Materials and methods

MeHgCl (98% purity) and DMeHg (95% purity) were obtained from Sigma-Aldrich (St. Louis, MO, USA). Mercury standard solution, formic acid and methanol were obtained from Nacalai Tesque Inc. (Kyoto, Japan). Na_2_S and XAD-4 were purchased from FUJIFILM Wako Pure Chemical Co. (Osaka, Japan) and ACROS Organics (Fair Lawn, NJ, USA), respectively. All other reagents used were of the highest purity available. (MeHg)_2_S was synthesized as previously described^16^.

### Animal experiments

All protocols for animal experiments, which were carried out in compliance with the ARRIVE guidelines, were approved by the University of Tsukuba Animal Care and Use Committee and followed the committee’s guidelines for alleviating suffering. C57BL/6 J 8-week-old female mice (Clea Japan, Tokyo, Japan) were fasted for 12 h before treatment with the compounds, and all experiments with exposure to chemicals were performed with a single administration.

After euthanasia, the intestines, intestinal contents and liver were removed for each experiment. The colon or duodenum was tied using a surgical suture before removal or washed with saline after removal. MeHg (200 µM in saline-5% methanol) was injected into the intestine, and then the intestine was covered by soaked Kimwipe with saline and incubated for 30 min at room temperature. The washed distal colon was incubated with MeHg (200 µM in saline-5% methanol) for 30 min at room temperature. The distal colon content (10 mg) was incubated with MeHg (200 µM) for 30 min at 25 °C. After incubation, formic acid (at a final concentration of 1%) was added to the mixture, which was then centrifuged at 14000*g* for 5 min. The supernatant was analyzed by HPLC/AAS as described below.

For the HPLC/AAS analysis of (MeHg)_2_S in intestinal contents or feces, mice were orally administered mercury compounds dissolved in saline. (MeHg)_2_S in the fecal samples from mice given 10 mg/kg MeHg for 18–42 h was extracted by 50% methanol-10% formic acid. After centrifugation (13000*g*, 4 °C), the samples were analyzed by HPLC/AAS as described below.

To detect DMeHg in the expirate, MeHgCl, DMeHg, or (MeHg)_2_S (0.1 mmol/kg) dissolved in corn oil was intraperitoneally injected into mice and then the expirate was aspirated by pumping (50 mL/min) through a mask connected to an XAD-4 (500 mg) column for 3 h. XAD-4 was set on both sides (air in/out) of the mask to avoid contamination with mercury species from the environment. Absorbed chemicals were extracted by 1 mL of acetone and analyzed by GC/MS (GCMS-QP2020, Shimadzu, Kyoto, Japan).

### Sample preparation using the mouse intestine contents and liver for HPLC/AAS

To determine the contribution of gut microbiota to (MeHg)_2_S formation from MeHg, the distal colon content was incubated at 25 °C or 95 °C for 15 min and then cooled on ice for 5 min. The content (10 mg) was incubated with MeHg [200 µM in phosphate-buffered saline (pH 7.5) (PBS)-5% methanol] for 30 min at 25 °C, followed by addition of formic acid (at a final concentration of 1%). Distal colon content (200 mg) from two mice for one experiment was homogenized in PBS (1 mL) and centrifuged at 9000*g*, the supernatant was collected, and then the low-molecular-weight fraction of the supernatant was fractionated using Amicon Ultra (Ultracel 3 K, Merck, Darmstadt, Germany) and the high-molecular-weight fraction was fractionated using a PD SpinTrap G-25 column (GE Healthcare, Buckinghamshire, UK). The reaction mixtures were centrifuged at 12000*g* for 5 min, and then the supernatant was analyzed by HPLC/AAS. An aliquot of homogenized distal colon content (200 mg/mL) was transferred into the principal chamber of a Thunberg tube, and PBS (1.75 mL) was added. Five hundred microliters of MeHg (1.2 mM in PBS-30% methanol) was transferred into another side of the Thunberg tube. The tube was vacuumed by a pump for 3 min and then filled with argon gas. This step was repeated three times to achieve anerobic conditions. Samples were mixed in the tube and incubated at 37 °C for 30 min. The reaction mixtures were centrifuged at 12000*g* for 5 min, and then the supernatant was analyzed by HPLC/AAS.

The mouse liver was homogenized in a 4× tissue volume of 50 mM KPi (pH 7.4), followed by centrifugation at 9000*g* for 10 min. The protein concentration of the 9000*g* supernatant was determined by a Protein Assay BCA Kit (Nacalai, Kyoto, Japan). (MeHg)_2_S (100 µM) was incubated in the liver supernatant centrifuged at 9000*g* (2 mg/mL) or in 50 mM KPi (pH 7.5) for 0–7 days at 37 °C, and liquid–liquid extraction was performed to determine the organic and/or inorganic Hg content by AAS (MA-3000; Nihon Instruments, Osaka, Japan). To determine the contribution of protein during the degradation of (MeHg)_2_S in the mouse liver 9000*g* supernatant, the 9000*g* supernatant (400 µg) was incubated at 25 °C or 95 °C for 15 min, followed by cooling on ice for 5 min. (MeHg)_2_S (at a final concentration of 100 µM) was incubated in the nonheated or heated supernatant (2 mg/mL) at 37 °C for 24 h. The low-molecular-weight fraction of 4 mg/mL of the liver 9000*g* supernatant was fractionated using Amicon Ultra (ultracel 3 K). (MeHg)_2_S (at a final concentration of 100 µM) was incubated in the low-molecular-weight fraction (2 mg/mL) at 37 °C for 24 h. After incubation, formic acid (at a final concentration of 1%) was added to the mixture, which was centrifuged at 14000*g* for 5 min. The supernatant was analyzed by HPLC/AAS.

### Analysis of MeHg and (MeHg)_2_S by HPLC/AAS

MeHg and (MeHg)_2_S in the fractions were detected with retention times of approximately 3–5 min and 10–15 min, respectively, as previously described^16,19^. Briefly, samples were subjected to HPLC equipped with a Zorbax Eclipse XDB-C_18_ column (50 mm long, 2.1 mm i.d., 5 µm particle size; Agilent Technologies, Santa Clara, CA, USA). The mobile phase was 10% methanol-0.1% formic acid (the flow rate was 0.5 mL/min), and the mercury concentrations in the eluate fractions were determined using a direct thermal decomposition mercury analyzer with an AAS detector (MA-3000). A mercury standard solution diluted in 100 mg/L cysteine solution was used to prepare a standard curve for AAS.

### Detection of organic and inorganic Hg from (MeHg)_2_S

(MeHg)_2_S (100 µM) in 50 mM KPi (pH 7.5) was incubated for 0–7 days at 37 °C. An aliquot of the sample (500 µL) was mixed with 100 µL of 6 N HCl and 600 µL of benzene and stirred for 5 min. After centrifugation (13000*g*, 5 min) of the mixture, a benzene layer was collected, and then 600 µL of benzene was added to the water layer. The extraction process was repeated four times. The Hg content was measured by AAS in the collected benzene layer and water layer, which was neutralized by the addition of 1.71 N NaOH (500 µL).

### Determination of insoluble mercury

(MeHg)_2_S (10 mM) in 50 mM KPi (pH 7.5) was stirred for 7 days at room temperature and then filtered. The residue was washed with 20 mL of pure water and 20 mL of methanol, dried using an aspirator, suspended in pure water and benzene and stirred for 5 min. After centrifugation, Hg in the benzene layer was determined by AAS. The process was repeated until the Hg content in the benzene layer was less than 10 ng. The solvents were then removed, and the residue was washed with 10 mL of methanol three times and dried by an aspirator. To determine the crystal structure of the compound, powder XRD experiments (Rigaku Ultima-III, Rigaku, Tokyo, Japan; Cu Kα, 40 kV–30 mA, θ/2θ scanning, angular step 0.02°, 3 s accumulation per step) were carried out.

### Detection of volatile Hg by GC/MS

Volatile Hg compounds were collected by XAD-4, and then the absorbed chemicals were extracted using 1 mL of acetone and analyzed by GC/MS. The samples were separated by an InertCap 5 MS/Sil column (30 m long, 0.25 mm i.d., 0.25 µm df; GL Sciences, Tokyo, Japan) that was maintained at 40 °C, with He (≥ 99.99995% purity) used as the carrier gas. The temperature was set as follows: 40 °C for 5 min; a linear increase to 150 °C (6 °C/min); hold at 150 °C for 5 min; and an increase to 200 °C over 1 min. The eluted compounds were then transferred to the EI source (70 eV) of the system, and the control and analyses were performed using GCMSsolution ver 4.44 (Shimadzu)*. *Unfortunately, we were not able to determine DMeHg quantitatively using GC/MS because of its high vapor pressure.

### Detection of CH_4_ by GC/MS

DMeHg (4 mM) or MeHgCl (4 mM) in 0.5 N HCl-50% methanol was incubated for 3 h or 4 days at room temperature in a glass vial, and then the sample was analyzed by GC/MS. Gas in the headspace of the sample tube was injected using a gas-tight syringe and separated by TC-BOND Q (30 m long, 0.32 mm i.d., 10 µm df; GL Sciences) with a PLOT column particle trap (2.5 m long, 0.25 mm i.d.; GL Sciences). The temperature was set as follows: 35 °C for 5 min; a linear increase to 220 °C (12 °C/min); and hold at 220 °C for 5 min. The eluted compounds were then transferred to the EI source of the system and analyzed using GCMSsolution (Shimadzu) as described above.

### Detection of MeHg by EI-MS

DMeHg (4 mM) in 0.5 N HCl-50% methanol was incubated for 4 days at room temperature in a glass vial. A 10 µL aliquot of the sample was subjected to EI-MS, and the control and treatment analyses were performed using GCMSsolution and direct-injection mode.

### Statistical analysis

Each experiment was repeated at least three times, and representative data are shown. All statistical analyses were performed using GraphPad Prism version 6.0 software (GraphPad, San Diego, CA, USA). Statistical significance was assessed by the differences in a population mean test, followed by the Welch test, or one-way ANOVA followed by a Tukey's multiple comparisons test with *P* < 0.05 considered significant.

## Supplementary Information

Supplementary Information.

## Data Availability

All data generated or analyzed during this study are included in this published article and its Supplementary Information files.
