# Supplementary material for: The fate of methylmercury through the formation of bismethylmercury sulfide as an intermediate in mice
Source: Sci Rep. 2021 Sep 2;11:17598. doi: 10.1038/s41598-021-96579-y (PMC8413320; doi:10.1038/s41598-021-96579-y)
Supplement: Supplementary file 1 — Supplementary Information. [file 41598_2021_96579_MOESM1_ESM.pdf]

Supplemental materials

The fate of methylmercury through the formation of bismethylmercury sulfide as an intermediate in mice

Yumi Abiko[1], Yusuke Katayama[2], Wenyang Zhao[3,4], Sawako Horai[5], Kenji Sakurai[3,4], Yoshito Kumagai[1, 2, *]

[1] Faculty of Medicine, University of Tsukuba, Tsukuba, Ibaraki 305-8575, Japan

[2] Master's Program in Medical Sciences, Graduate School of Comprehensive Human Sciences, University of Tsukuba, Tsukuba, Ibaraki 305-8575, Japan

[3] Doctoral Program in Materials Science and Engineering, Graduate School of Pure and Applied Sciences, University of Tsukuba, Tsukuba, Ibaraki 305-0006

[4] National Institute for Materials Science, Tsukuba, Ibaraki 305-0047, Japan

[5] Environmental Health Section, Dept. Environment and Public Health, National Institute for Minamata Disease, Minamata, Kumamoto 867-0008, Japan

[*]Address correspondence to Yoshito Kumagai, Faculty of Medicine, University of Tsukuba, 1-1-1 Tennodai, Tsukuba, Ibaraki 305-8575, Japan; Tel and Fax, +81-29-853-3133; E-mail, yk-em-tu@md.tsukuba.ac.jp

CONTENTS

Experimental Procedure

Figure S1

Figure S2

Figure S3

Reference

Experimental procedure

Detection of $(MeHg)_2S$ in the intestinal content of a mongoose

A wild small Indian mongoose (*Herpestes auropunctatus*) was collected at Goga, Nago city, Okinawa Prefecture, in 2017 as part of a wildlife damage-control program[1], and the intestinal content of the mongoose was mixed with a 5 volume of 50% methanol-10% formic acid to extract $(MeHg)_2S$. After centrifugation (20,000 *g*, 10 min, 4°C) of the mixture, the supernatant was analyzed by HPLC/AAS.

Figure S1

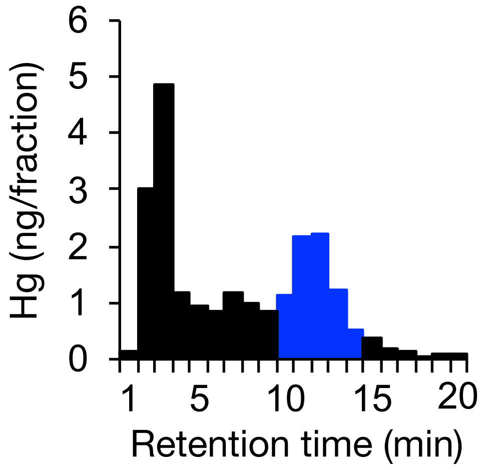

Figure S1. Analysis of $(MeHg)_2S$ in the intestinal content of mongoose. The blue bars indicate $(MeHg)_2S$. $(MeHg)_2S$ in the intestinal content of the mongoose was extracted by 50% methanol-10% formic acid, and then the supernatant was analyzed by HPLC/AAS. The blue bar indicates $(MeHg)_2S$, and representative data are shown.

Figure S2

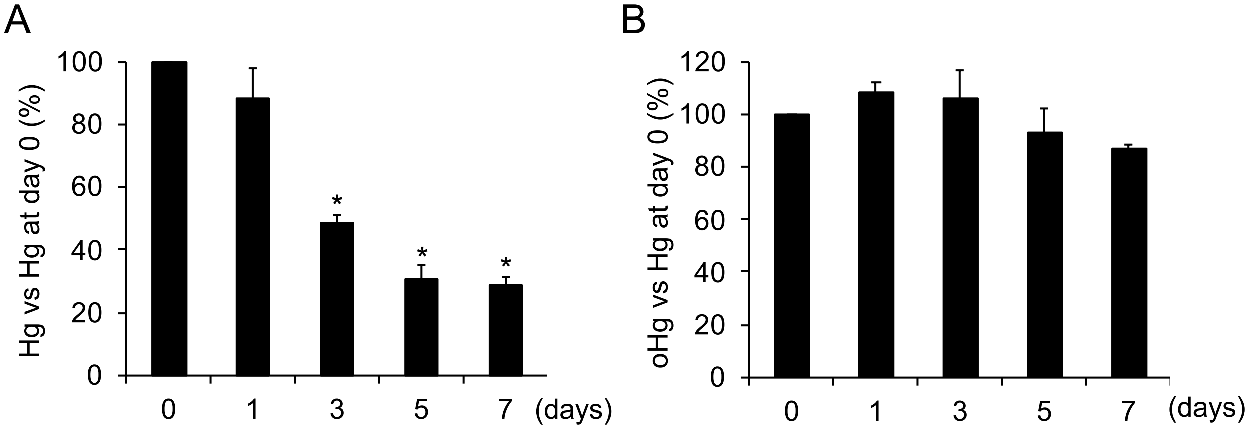

Figure S2. Detection of Hg during incubation of $(MeHg)_2S$ in KPi buffer.

(A) $(MeHg)_2S$ (100 μM) in 50 mM KPi (pH 7.5) was incubated for 0–7 days at 37°C, and the Hg content was measured by AAS. (B) MeHg in 50 mM KPi (pH 7.5) was incubated for 0–7 days at 37°C. After liquid-liquid extraction, the benzene layer and water layer were separately analyzed by AAS. The mercury content in the benzene layer was determined because the Hg level in the water layer was negligible. Each value is the mean ± SE of three determinations. *$P < 0.05$ vs. day 0.

Figure S3

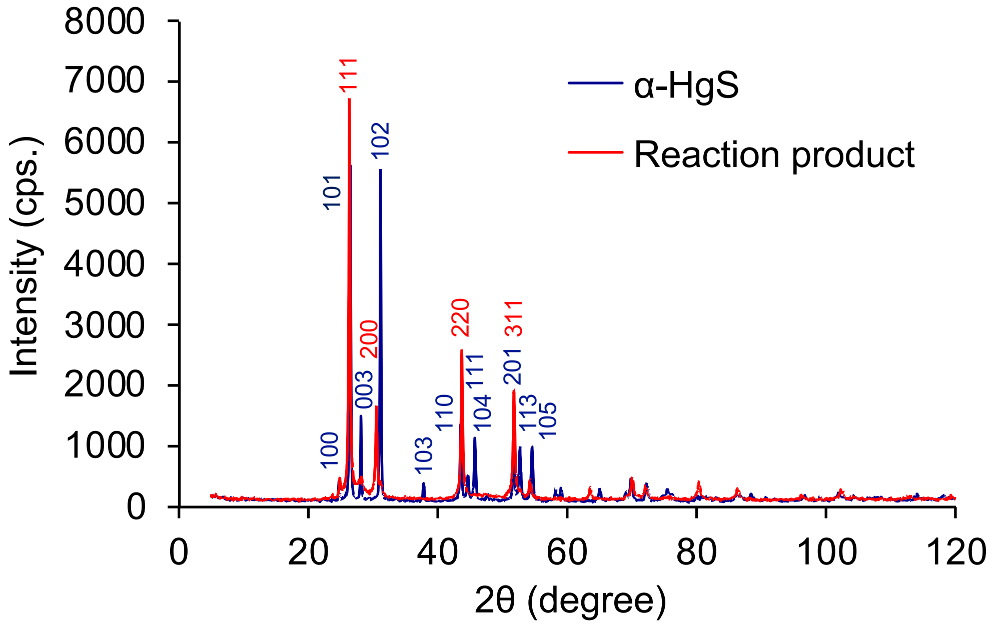

Figure S3. Analysis of insoluble mercury compounds during incubation with $(MeHg)_2S$. $(MeHg)_2S$ in 50 mM KPi (pH 7.5) was incubated for 7 days, and the reaction product in the water layer was purified. The reaction product (red) and authentic α-HgS (blue) were analyzed by X-ray diffraction, and the spectra are shown.

There are three small suspicious peaks at 24.84 deg, 28.16 deg, and 31.22 deg in the XRD pattern of the reference β-HgS (see Figure 3H) and the reaction product. One assumption could be that they are 100, 101, and 102 reflections of α-HgS. Under this assumption, α-HgS may be present in the black particles and even in the reference β-HgS sample as a secondary minor phase. However, considering the very weak peak intensities, this secondary minor phase is negligible. The conclusion of the present study would not be affected

Reference

1 Horai, S. *et al.* Establishment of a primary hepatocyte culture from the small Indian mongoose (Herpestes auropunctatus) and distribution of mercury in liver tissue. *Ecotoxicology* **23**, 1681-1689, doi:10.1007/s10646-014-1307-6 (2014).
